# Our New York Letter

**Published:** 1887-11

**Authors:** 


### Correspondence.

### OUR NEW YORK LETTER.

POISONING BY ILLUMINATING GAS TREATED BY RECTAL INJECTIONS OF OXYGEN—POVERTY OF GENERAL GRANT'S PHYSICIAN—A NEW HOSPITAL FOR CHILDREN—THE LOOMIS LABORATORY—OPENING OF THE NEW COLLEGE OF PHYSICIANS AND SURGEONS—ASIATIC CHOLERA ON THE STEAMSHIP ALESIA.

*Editors Atlanta Medical and Surgical Journal:*

The Bergeon method of using gaseous enemata as a therapeutic measure has probably disappointed the hopes of many experimenters in the treatment of pulmonary consumption, for to all accounts the average results obtained by this method are hardly as brilliant as we were led to believe might be expected. But even if this method fails to cure phthisis, it appears to be of considerable value still in another direction.

At St. Vincent's Hospital, in this city, a patient was admitted, suffering from poisoning by illuminating gas. He was in an unconscious condition and appeared to be almost moribund. It was decided to use the Bergeon apparatus for administering sulphureted hydrogen in this patient, but to substitute oxygen gas. This was done, and in about fifteen minutes signs of improvement were visible and the case went on to a good recovery. Another patient, suffering from gas-poisoning, and in a very similar condition to the first, was treated in a like manner and with a very happy termination. Since these results were obtained, the same plan of treatment was carried out on a patient at Bellevue Hospital, who had remained unconscious for about twenty-four hours. After administering the oxygen by the rectum, he soon began to improve. Should this treatment prove, after thorough testing, to be efficacious in this trouble, it must certainly be considered a valuable discovery, as cases of gas-poisoning are too common in cities, and so far a large percentage of them have failed to recover.

For sometime it has been rumored that Dr. John H. Douglass, who attended General Grant throughout his last illness, has become very much reduced in circumstances financially, and that his practice has materially suffered thereby. This appears to be true to a certain extent only. His practice has dropped off considerably, but he cannot be truly considered a poor man yet. It also appears that he received from the Grant family, for professional services rendered to the General, the full amount for his bill, which was about $7,000. I have heard that he was also presented with $5,000 as a special gift, which the General wished him to accept in addition to the amount of his bill. It is the intention of Dr. Douglass to publish very soon an account of his professional services to General Grant, including letters and other communications from the General to the doctor, and also different interesting conversations which took place between them.

A number of kindly-disposed and prominent ladies of this city have decided to organize a hospital for babies, where children of any age or suffering from any disease may be treated.

A corner building on Murray Hill has been selected for this purpose, which will accommodate one hundred patients. Already sufficient money has been raised to pay the running expenses for a year, and there is every prospect of the institution having a successful future. Among the physicians who will be connected with this hospital may be mentioned Drs. Andrew H. Smith, Thos. E. Satterthwaite, M. A. Starr, F. Kinnicutt and others.

Opposite Bellevue Hospital, on 26th street, is a building in process of construction which is nearly completed. This will be known as the Loomis Laboratory, and it is one of the latest additions to the Medical Department of the New York University Certain friends of Dr. Alfred L. Loomis have donated $100,000, which pays the cost of this structure, but the donors do not wish their names made public.

For a long time complaints have been heard at the Bureau of

Vital Statistics of the careless way in which physicians report their cases of births and contagious diseases, and it has been estimated that a large percentage of these cases are never registered. In view of securing more accurate records, it has been determined to remunerate all physicians for whatever outlay in postage stamps is necessary to report child-birth, and already blanks for reporting contagious diseases, printed on postal cards, have been prepared and may be had on application. It is now expected that the law will be strictly obeyed, or speedy prosecution may be expected to follow.

Since my last communication, the new quarters of the College of Physicians and Surgeons have been formally opened. The opening exercises which took place in the afternoon, were held in the principal lecture-room and were attended by a very large gathering of medical celebrities from this and other cities, besides a host of students and others. On the platform, besides the college faculty, were Cornelius and George Vanderbilt and Wm. D. Sloane. These gentlemen have been most generous in their donations, and the college is almost as much under obligations to them as to their deceased relative. The exercises were opened with prayer by Dr. Hardy, the college chaplain, after which the President, Dr. John C. Dalton, made the opening address. He described in a very vivid and interesting manner the career of this institution from its beginning in 1807, through its seasons of adversity and prosperity, down to the present time, and ended his remarks by paying a very handsome eulogy to the college benefactor, the late Mr. Wm. H. Vanderbilt. It was intended that Mr. Joseph H. Choate should deliver the inaugural address, but sickness in his family prevented this and Dr. Wm. H. Draper took his place. In his usual eloquent manner he spoke of the low standard of medical education in this country, compared with that of England and the continent of Europe, but he said it had always been the policy of the college to narrow the gate by which its graduates passed out, and lately it had been determined to narrow also the portal of entrance. At the end of this address Dr. Draper presented the college with portraits of Dr. Samuel L. Mitchell, Dr. Hooper Hosack, Dr. John C. Dalton and a bronze bust of Mr. Wm. H. Vanderbilt. In the evening the Alumni Association held a banquet at Delmonico's and a large attendance were present. Dr. C. R. Agnew presided, and although a number who were expected failed to come, the evening was passed in a thoroughly enjoyable manner.

It is said to be about fourteen years since we had cholera brought into this country, but at present the French steamship, Alesia, on which sixteen cases of Asiatic cholera were discovered, is being detained at quarantine in the lower bay of New York. This vessel sailed from Naples with over 600 passengers, and when nine days out, the disease began to show itself and eight died during the passage and were buried at sea. Arriving in this port, the sick passengers were transferred to the hospital on Swinburne Island, and the others were sent to Hoffman Island to be kept under observation until all danger is over. At the time of writing eleven more persons have died at the quarantine station, but the physicians in charge here feel confident that the disease is under control, and the ship and cargo, besides passengers' clothing, will all be subjected to a thorough process of disinfection before leaving present quarters. A. F.

*October, 1887.*

